# Full-Exon Pyrosequencing Screening of BRCA Germline Mutations in Mexican Women with Inherited Breast and Ovarian Cancer

**DOI:** 10.1371/journal.pone.0037432

**Published:** 2012-05-24

**Authors:** Felipe Vaca-Paniagua, Rosa María Alvarez-Gomez, Verónica Fragoso-Ontiveros, Silvia Vidal-Millan, Luis Alonso Herrera, David Cantú, Enrique Bargallo-Rocha, Alejandro Mohar, César López-Camarillo, Carlos Pérez-Plasencia

**Affiliations:** 1 Laboratorio de Genómica, Instituto Nacional de Cancerología, Tlalpan, México; 2 Unidad de Genómica y Secuenciación Masiva (UGESEM), Instituto Nacional de Cancerología, Tlalpan, México; 3 Clínica de Genética, Instituto Nacional de Cancerología, Tlalpan, México; 4 Unidad de Investigaciones Biomédicas en Cáncer, Instituto Nacional de Cancerología, Instituto de Investigaciones Biomédicas, Universidad Nacional Autónoma de México (UNAM), Tlalpan, México; 5 Departamento de Oncología Médica, Instituto Nacional de Cancerología, Tlalpan, México; 6 Posgrado en Ciencias Genómicas, UACM, Benito Juarez, México; 7 Unidad de Biomedicina, FES-IZTACALA, UNAM, Tlalnepantla, México; Cedars-Sinai Medical Center, United States of America

## Abstract

Hereditary breast cancer comprises 10% of all breast cancers. The most prevalent genes causing this pathology are *BRCA1* and *BRCA2* (*breast cancer early onset 1* and *2*), which also predispose to other cancers. Despite the outstanding relevance of genetic screening of BRCA deleterious variants in patients with a history of familial cancer, this practice is not common in Latin American public institutions. In this work we assessed mutations in the entire exonic and splice-site regions of BRCA in 39 patients with breast and ovarian cancer and with familial history of breast cancer or with clinical features suggestive for BRCA mutations by massive parallel pyrosequencing. First we evaluated the method with controls and found 41–485 reads per sequence in BRCA pathogenic mutations. Negative controls did not show deleterious variants, confirming the suitability of the approach. In patients diagnosed with cancer we found 4 novel deleterious mutations (c.2805_2808delAGAT and c.3124_3133delAGCAATATTA in *BRCA1*; c.2639_2640delTG and c.5114_5117delTAAA in *BRCA2*). The prevalence of BRCA mutations in these patients was 10.2%. Moreover, we discovered 16 variants with unknown clinical significance (11 in exons and 5 in introns); 4 were predicted as possibly pathogenic by *in silico* analyses, and 3 have not been described previously. This study illustrates how massive pyrosequencing technology can be applied to screen for BRCA mutations in the whole exonic and splice regions in patients with suspected BRCA-related cancers. This is the first effort to analyse the mutational status of BRCA genes on a Mexican-mestizo population by means of pyrosequencing.

## Introduction

About 10% of all breast cancers are of monogenic origin [1]. The most prevalent entity is Hereditary Breast and Ovarian Cancer (HBOC), an autosomal dominant disease with incomplete penetrance. The two high-penetrance genes most commonly mutated in HBOC are the tumor suppressor genes *BRCA1* and *BRCA2* (*breast cancer, early onset 1* and *2*). The *BRCA1* gene, localized at 17q21, and *BRCA2*, at 13q12, have long coding sequences (5589 and 10254 nt for *BRCA1* and *BRCA2*, respectively) and are essential components of the double-strand break repair by homologous recombination system [2]. Almost 3500 deleterious mutations in these genes have been found in all the coding sequence [3]. Furthermore *BRCA1* and *BRCA2* mutation carriers are also at increased risk of fallopian tubes, pancreatic, prostate and endometrial cancer [4]–[6].

The molecular diagnosis of mutations in *BRCA* genes implies high degree of clinical suspicion based principally in history of familial BRCA-related cancers in first- or second-degree relatives, age of presentation and tumor characteristics (morphological, immunohistochemical and molecular features) [7]. For patients with a BRCA mutation, current clinical alternatives include breast and ovarian screening, prophylactic surgery, and chemoprevention [8]. The approach extends to their family in order to identify other members at risk to allow the genetic advice, screening and/or predictive testing [9].

Unfortunately, genetic testing for mutations in *BRCA1* and *BRCA2* is not always available in public institutions in developing countries due to its high cost and limitations in infrastructure. As BRCA genes have long coding sequences and lack mutation hot spots, the current strategies for BRCA genotyping typically include a first step to detect occurring mutations by protein truncation test (PTT), denaturing high-performance liquid chromatography (dHPLC), denaturing gradient gel electrophoresis (DGGE) or high-resolution melting curve analysis (HRMCA); and a final step to determine the mutation by Sanger sequencing [10]. These approaches are laborious, expensive and time consuming, and could be substituted by high throughput, cost efficient testing methods such as massively parallel sequencing [11], [12].

In this work we used massive parallel pyrosequencing to screen for mutations in the complete coding regions and splice sites of *BRCA* genes in Mexican women. We studied 39 patients with breast and/or ovary cancer and with history of familial cancer and with early-onset breast cancer, suggestive for *BRCA* mutations. We found 4 pathogenic mutations, of which 3 have not been described. We also identified 16 missense mutations with unknown deleterious effects. In addition, by a directed sequencing strategy, we evaluated the presence of the deleterious mutations in the family members of the patients. Also, we identified family members with the mutations and with no clinical manifestations of cancer. These patients began clinical management (that includes follow-up and prophylactic measures). This work illustrates how new sequencing technology for screening of mutations in *BRCA* genes impacts the familial health scenario and can be conducted as part of the genetic approach for patients with familial cancer in public health care institutions.

## Methods

### Patients

A total of 39 patients were screened. Thirty-five female patients with breast and/or ovarian cancer and with two or more first- or second-degree relatives with tumors associated with *BRCA* mutations were studied. Two male patients with breast cancer were included. All patients were clinically approached and a three-generation genealogy of each family was made. Two patients without familial cancer history, one with early-onset (age of diagnosis: 28) breast cancer and one with breast and ovarian cancer, suggestive for *BRCA* mutations, were also included. Patients were fully informed about the study and gave their written consent. The protocol was approved by the Institutional Review Boards of the National Cancer Institute of Mexico (http://www.incan.edu.mx/) and carried out in accordance with the Declaration of Helsinki, good clinical practices, and local ethical and legal requirements.

### DNA isolation

Genomic DNA was isolated of peripheral blood with the Magna Pure System (Roche) following manufacturer instructions. The integrity of the material was verified by agarose electrophoresis. Sample quantification was done with the Quant-it Picogreen kit (Invitrogen) in a QuantiFluor Fluorometer (Promega).

### Pyrosequencing

A Sequencing Master library of amplicons covering all the coding exons and splice sites of *BRCA1* and *BRCA2* was produced for each patient using the BRCAMASTR kit (Multiplicom) following manufacturer instructions. Briefly, 50 ng of gDNA were used as template in each of 12 multiplex PCR reactions for each patient. These reactions amplified the complete exonic and splice sites of *BRCA1* and *BRCA2*. A 1∶1000 dilution of the purified PCR products were re-amplified using molecular identification (MID) adaptors for each patient. A BRCA amplicon library of each patient was generated and equivalent concentrations of the libraries were pooled to generate a Sequencing Master library. Pyrosequencing of the Master libraries were done in the sense and anti-sense strands with the 454 GS Junior (Roche) technology. Data analysis was done with the GS Amplicon Variant Analyzer software (Roche) comparing against genomic references NG_005905 and NG_012772 for *BRCA1* and *BRCA2*, respectively. The cDNA references utilized were NM_007294 and NM_000059 for *BRCA1* and *BRCA2*, respectively. The nomenclature used is based on the cDNA sequence and is according to Human Genome Variation Society (http://www.hgvs.org/). All the deleterious mutations found were verified by Sanger sequencing of original patient blood DNA and by restriction analysis when possible. The putative functional effects of missense variants were analyzed *in silico* with PolyPhen-2 (http://genetics.bwh.harvard.edu/pph2/).

### Restriction analysis

The presence of the mutation c.3124_3133delAGCAATATTA found in patient 11 was verified by restriction analysis of the PCR product (554 pb) amplified with the primers BRCA1-11.1F: TCAGAGGCAACGAAACTGGACTCA and BRCA1-11.1R: CAGCCTATGGGAAGTAGTCATGCA. The mutated allele lacks the restriction site for *Ssp*I (AATATT) and is not cleaved by this enzyme, while the wild-type allele is cleaved in two fragments (257 and 297 pb). 500 ng of PCR products were digested with 1 U of *Ssp*I (Fermentas) at 37°C for 4 h in 20 uL. Ten uL of the reactions were visualized in 1.5% agarose gels.

## Results

**Figure 1:**
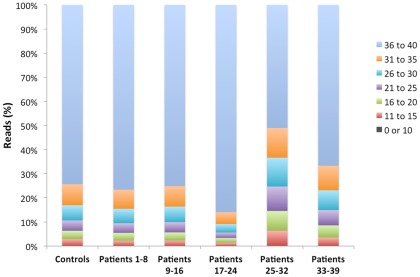
Quality of the sequencing runs. The percentages of the reads with their associated quality numbers of all runs are plotted.

**Figure 2:**
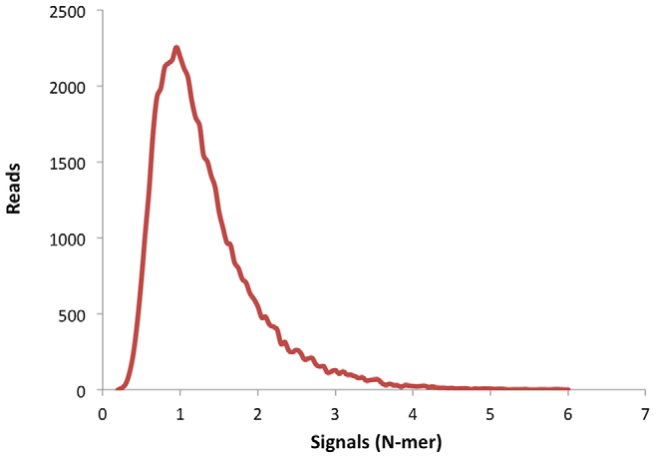
Distribution of homopolymeric tracts across the reads. The base number signals are plotted against the sequence reads of the control run.

To analyze the performance of the amplicon strategy for the sequencing of BRCA genes we carried out an evaluation run with 6 patients' samples, of which 4 had previously identified mutations and 2 were negative controls [13]. We used three inclusion criteria to accept valid mutated sequences: 1) mutation found in forward and reverse sequences, 2) at least 30% of sequences with the mutations and 3) at least 20X of sequence coverage of the amplicons with the mutation. Also we defined three exclusion criteria: 1) mutations detected in an homopolymeric tract of ≥6, 2) mutations found in the last nucleotide of the sequence and with frequencies of less than 30% and 3) quality score lower than 20 in forward and reverse reads. Similar criteria have been described elsewhere [12]. As seen in table 1, we detected all the deleterious mutations in the positive controls and no pathogenic variants were found in the negative controls. In the mutations observed the minimal and maximal coverage was 41 and 485 reads per nucleotide, respectively. Also in this control experiment more than 70% of all the reads across the whole exon and splice sites had a quality score (Q) ranging from 36 to 40 (highest score), and low quality reads with Q>20 were less than 10% (Fig. 1). As expected, we observed that the majority of these low quality reads were in homopolymeric tracts, especially of >6 bases. Although present, these homopolymeric sequences are a negligible number of the total reads (Fig. 2). With this analysis we concluded that the strategy used was robust and suitable for its application in the screening of BRCA mutations in patients' samples.

**Table 1:** Evaluation of the methodological strategy for the detection of BRCA mutations.

Sample	Gene	Deleterious Mutation	Type of mutation^a^	Position (aa)	Stop codón position (aa)	Coverage^1^	Clinical relevance	BIC reported	Reference
Control (+)1	*BRCA1*	c.4065_4068delTCAA	F	1355	1364	41	Yes	Yes	[13], [47]–[49]
Control (+)2	*BRCA2*	c.2808_2811delACAA	F	936	958	459	Yes	Yes	[50]
Control (+)3	*BRCA2*	c.9382C>T	S	3128	3128	485	Yes	Yes	[51], [52]
Control (−)1	-	None detected	-	-	-	-	-	-	-
Control (−)2	-	None detected	-	-	-	-	-	-	-

1 Number of reads per nucleotide.

a Types of mutations: F: frameshift; S: stop.

**Figure 3:**
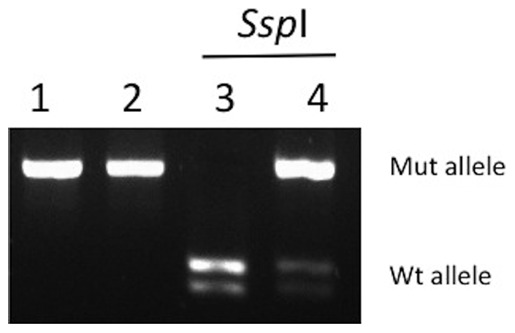
Restriction analysis of the mutation c.3124_3133delAGCAATATTA found in patient 3. PCR products encompassing the mutation were digested with *Ssp*I (see methods). The mutated allele has lost the *Ssp*I site and is not cleaved by the enzyme, while the wild-type allele is cut in two fragments. Lanes: 1) wild-type control PCR product not digested, 2) patient 11 PCR product not digested, 3) wild-type control PCR product digested, 4) patient 11 PCR product digested. Mut: mutated; Wt: wild-type.

**Figure 4:**
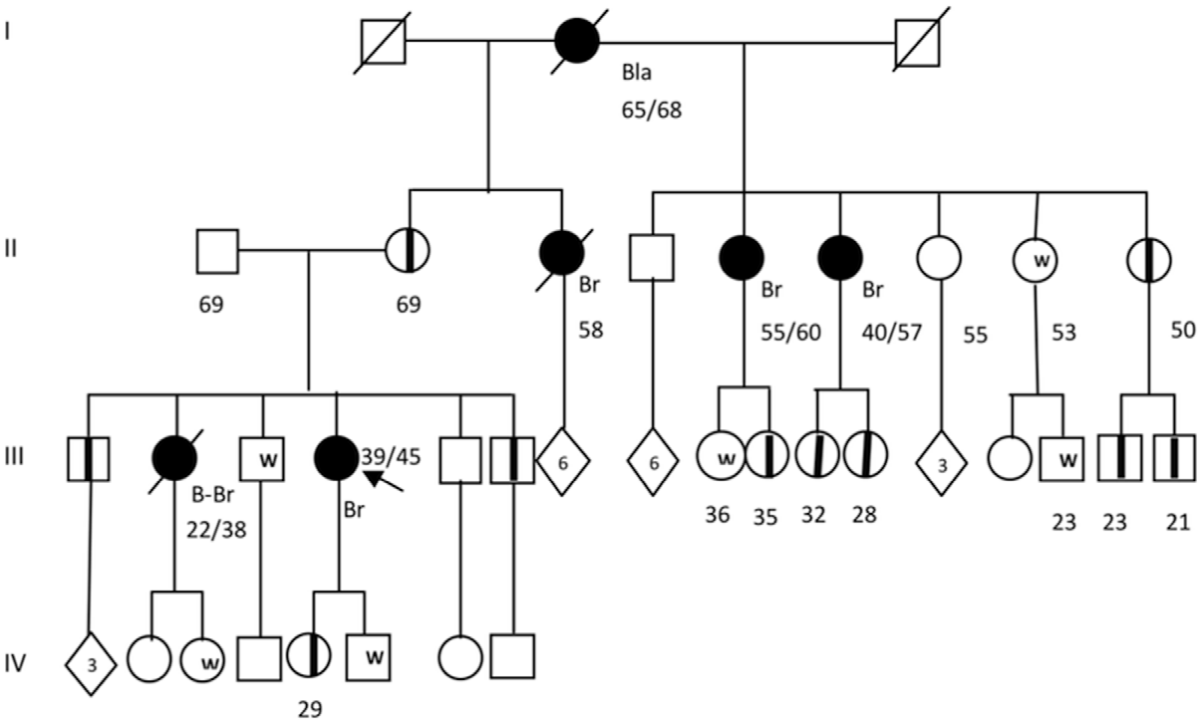
Genealogy of the family 1 carrier of the deleterious mutation c.5114_5117delTAAA in *BRCA2*. Index patient is denoted with an arrow. Individuals with cancer are represented with in dark circles or with dark squares; the type of cancer is indicated as follows: Bla: Bladder cancer; Br: Unilateral Breast Cancer; B-Br: Bilateral breast cancer. Current age or known ages of cancer diagnosis and decease are showed. Numbers inside the rhombi indicate quantity of relatives. Asymptomatic carriers are represented with a midline. Unaffected family members confirmed by the predictive molecular testing are shown with a W (wild type).

**Figure 5:**
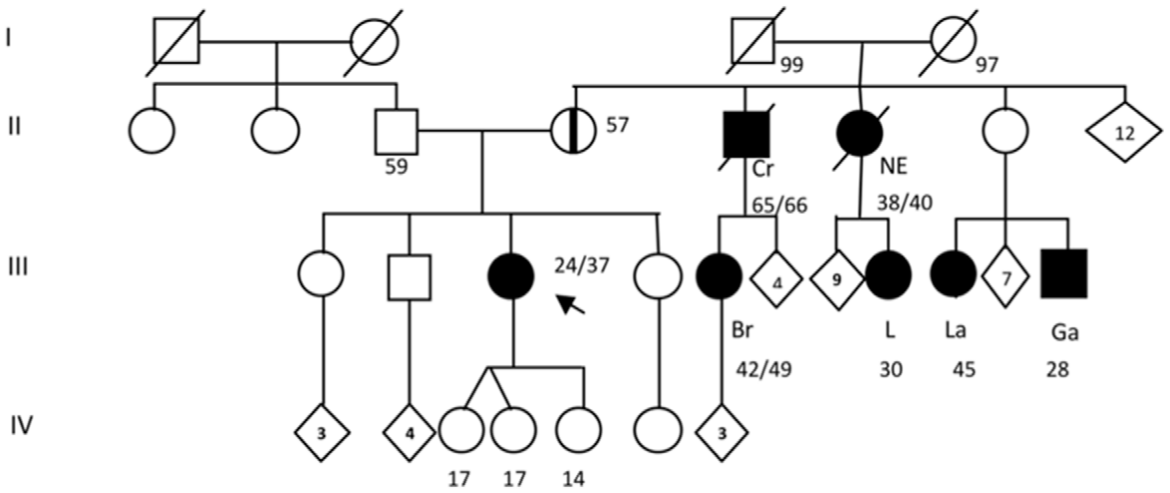
Genealogy of the family 15 carrier of the deleterious mutation c.2639_2640delTG in *BRCA2*. Individuals with cancer are represented with dark circles or with dark squares; the type of cancer is indicated as follows: Br: unilateral breast cancer; Cr: colorectal cancer; NE: Not especified neoplasia; L: lung cancer; La: laryngeal cancer; Ga: gastric cancer. Index patient is denoted with an arrow. Current age or known ages of cancer diagnosis and decease are showed. Numbers inside the rhombi indicate quantity of first-degree relatives. Asymptomatic carriers are represented with a midline.

**Figure 6:**
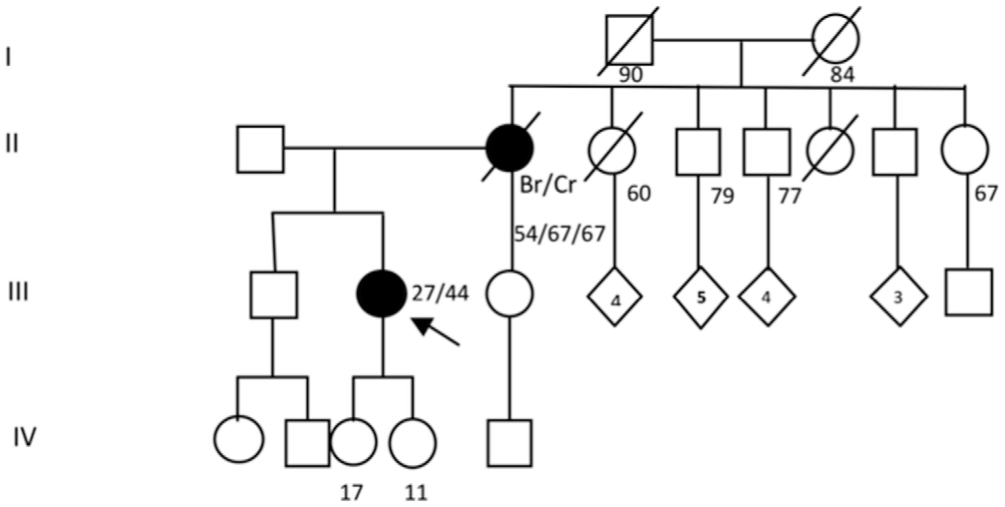
Genealogy of the family 39 carrier of the deleterious mutation c.2805_2808delAGAT in *BRCA1*. Index patient is denoted with an arrow. Individuals with cancer are represented in dark; the type of cancer is indicated as follows: Br: unilateral breast cancer; Cr: colorectal cancer. Current age or known ages of cancer diagnosis and decease are showed. Numbers inside the rhombi indicate quantity of relatives.

We screened for mutations in the whole coding sequence of BRCA genes in 39 patients with early-onset breast and ovarian tumors and/or with familial history of cancer, suggestive for *BRCA* mutations, as determined by our Clinic of Genetics. The main clinical characteristics of the patients are listed in table 2 and 3. After the pyrosequencing analysis and careful examination of the reads with our criteria of inclusion and exclusion, we found 4 mutations in the BRCA genes (c.2805_2808delAGAT and c.3124_3133delAGCAATATTA in *BRCA1*; c.2639_2640delTG and c.5114_5117delTAAA in *BRCA2*). All mutations were predicted to be deleterious because each generated a stop codon in the open reading frame (Table 4). These pathogenic mutations were confirmed by Sanger sequencing and the c.3124_3133delAGCAATATTA mutation in *BRCA1* was also confirmed by restriction analysis (Fig 3). In the family of patient 1 (mutation c.5114_5117delTAAA) we found 10 clinically asymptomatic carriers (Fig. 4). The family with the c.2639_2640delTG mutation in *BRCA2* (patient 15) had a strong history of cancer, including laryngeal, gastric, lung and colon cancer in second- and third-degree relatives in the maternal branch (Fig. 5). In the family with the c.2805delAGAT mutation in *BRCA1* (patient 39), one first-degree relative had breast and colon cancer (Fig. 6). Interestingly, 3 of the 4 deleterious mutations have not been described previously. Likewise, we detected 16 genetic variants with unknown clinical significance (VUS), which included missense mutations and changes in intronic sequences (Table 5). Four VUS were predicted to be potentially deleterious by *in silico* analyzes (Table 5). Intronic variants that have been evaluated functionally through *in vitro* experiments by others were not present [14]. No Ashkenazi founder mutations were found.

**Table 2:** Clinical features of the patients with *BRCA* mutations.

Sample	Age (years)	Cancer Type	Age diagnosis (years)	Familial cancer history	Tumor Histological Features	Other Tumor Features a
Patient 1	31	Breast cancer	31	Yes	Canalicular carcinoma	ER positive, PR positive, Her2/neu positive
Patient 3	42	Ovarian cancer	33	No	Ovarian serous adenocarcinoma	Not reported
		Unilateral Breast cancer	38		Canalicular carcinoma	Triple negative
Patient 15	37	Ovarian cancer	24	Yes	Ovarian serous adenocarcinoma	Not reported
		Unilateral breast cancer (right)	37		Canalicular carcinoma, brisk lymphocytic infiltrate	ER positive, PR positive and Her2/neu negative Ki-67: 5%
Patient 39	44	Bilateral breast cancer	27	Yes	Canalicular carcinoma	Triple negative

a ER  =  estrogen receptor; PR  =  progesterone receptor; HER2/neu  =  human epidermal growth factor receptor 2; Ki-67 =  antigen KI 67.

**Table 3:** Clinical and familial features of the patients included in the study.

Sample	Age (years)	Gender	Cancer Type	Tumor Histological Features	Other Tumor Features	Age diagnosis (years)	Familial cancer history	Family members with cancer			
								Number	Tumor type	Degree	Youngest age at diagnosis (years)
Patient 1	31	Female	Breast cancer	Canalicular carcinoma	ER (+), PR (+) and Her2/neu (+)	31	Yes	5	Bilateral BC, Unilateral BC Bladder cancer	1^st^, 2^nd^ and 3^rd^	22
Patient 2	35	Female	Unilateral Breast cancer	Canalicular carcinoma	ER (+), PR (+) and Her2/neu (−)	30	Yes	5	Breast cancer. Colorectal cancer	1^st^ and 2^nd^	36
Patient 3	42	Female	Ovarian cancer	Ovarian serous adenocarcinoma	Not reported	33	No				
			Unilateral Breast cancer	Canalicular carcinoma	Triple negative	38					
Patient 4	40	Female	Unilateral breast cancer	In situ, canalicular carcinoma	ER (+), PR (+) and Her2/neu (−)	38	Yes	3	Breast cancer	1^st^ and 2^nd^	42
Patient 5	64	Female	Unilateral breast cancer	Canalicular carcinoma	ER (+), PR (+)	63	Yes	2	Breast cancer	1^st^	51
Patient 6	66	Female	Unilateral breast cancer	Canalicular carcinoma	ER (+), PR (+)	53	Yes	10	Breast cancer. Ovarian cancer. Lymphoma. Intestinal cancer.	1^st^, 2^nd^ and 3^rd^.	40
Patient 7	29	Female	Unilateral breast cancer	Canalicular carcinoma	Triple negative	28	Yes	5	Breast, pancreatic, colorectal and bladder cancer	2^nd^	47
Patient 8	48	Female	Breast cancer	Canalicular carcinoma	NA	47	Yes	2	Breast cancer. Ovarian cancer.	1^st^	41
Patient 9	42	Female	Breast cancer	Canalicular carcinoma	NA	41	Yes	2	Breast cancer. Ovarian cancer	1^st^	45
Patient 10	68	Female	Breast cancer	Canalicular carcinoma	ER (+), PR (+)	60	Yes	13	Breast, pancreatic, lung, liver and colorectal cancer	2^nd^ and 3^rd^	44
Patient 11	53	Female	Unilateral breast cancer	Canalicular carcinoma	ER (+), PR (+) and Her2/neu (−)	49	Yes	13	Breast, pancreatic, lung, liver and colorectal cancer	2^nd^ and 3^rd^	44
Patient 12	68	Male	Unilateral breast cancer and colorectal cancer	Breast: Canalicular carcinoma.	NA	Colorectal cancer: 52 Breast cancer: 56	Yes	1	Breast cancer	1^st^	39
Patient 13	62	Female	Bilateral breast cancer	Multifocal, canalicular carcinoma	ER (+), PR (+) and Her2/neu (−)	1^st^: 48, 2^nd^: 60	Yes	5	Breast, ovarian and skin cancer	1^st^ and 2^nd^	36
Patient 14	37	Female	Unilateral breast cancer	Canalicular carcinoma	Triple negative	35	Yes	6	Breast, prostatic and renal cancer	1^st^ and 2^nd^	28
Patient 15	37	Female	Ovarian cancer	Ovarian serous adenocarcinoma	Not reported	24	Yes	6	Breast, laryngeal, lung, gastric and colorectal.	2^nd^ and 3^rd^	28
			Unilateral breast cancer (right)	Canalicular carcinoma, brisk lymphocytic infiltrate	ER (+), PR (+) and Her2/neu (−)	37					
Patient 16	30	Female	Unilateral breast cancer	Canalicular carcinoma	ER (+), PR (+) and Her2/neu (−)	28	Yes	1	Abdominal cancer (NA)	1^st^	31
Patient 17	33	Female	Unilateral breast cancer	Canalicular carcinoma	ER (+), PR (+) and Her2/neu (−)	30	Yes	1	Breast and ovarian cancer	1^st^	55
Patient 18	42	Female	Bilateral breast cancer	Canalicular carcinoma	Triple negative (both tumors)	1st: 34, 2nd: 39	Yes	1	Breast cancer	1^st^	41
Patient 19	39	Female	Unilateral breast cancer	Canalicular carcinoma	Triple negative	36	Yes	7	Breast cancer	1^st^, 2^nd^ and 3^rd^	<40 (NA)
Patient 20	65	Male	Unilateral breast cancer	Lobulillar carcinoma	Triple negative, androgen negative	63	Yes	1	Ovarian cancer	1^st^	44
Patient 21	28	Female	Unilateral breast cancer	Canalicular carcinoma	Triple negative	27	Yes	2	Laringeal cancer and abdominal caáncer (NA)	2^nd^	50
Patient 22	35	Female	Unilateral breast cancer	Canalicular carcinoma	Triple negative	32	Yes	2	Prostatic cancer	2^nd^	58
Patient 23	33	Female	Unilateral breast cancer	Canalicular carcinoma	ER (+), PR (+) and Her2/neu (+)	32	NA (no contact with family)	NA	NA	NA	-
Patient¶24	58	Female	Unilateral breast cancer	Canalicular carcinoma	ER (−), PR (−) and Her2/neu (+)	56	Yes	2	Breast and colorectal cancer	1^st^	33
Patient 25	48	Female	Unilateral breast cancer	Canalicular carcinoma	NA	37	Yes	2	Breast and gastric cancer	2^nd^	40
Patient 26	31	Female	Unilateral breast cancer	Canalicular carcinoma	ER (+), PR (+) and Her2/neu (+)	29	Yes	4	Skin, laringeal and intestinal cancer	2^nd^ and 3^rd^	35
Patient 27	35	Female	Unilateral breast cancer	Canalicular carcinoma	Triple negative	33	Yes	1	Abdominal cancer (NA)	2^nd^	30
Patient 28	30	Female	Unilateral breast cancer	Canalicular carcinoma	Triple negative	25	Yes	4	Brest, gastric and thyroid cancer	2^nd^	30
Patient 29	53	Female	Bilateral breast cancer	Canalicular carcinoma	Triple negative	52	Yes	5	Bilateral and unilateral breast cancer. Liver cancer	1^st^, 2^nd^ and 3^rd^	40
Patient 30	25	Female	Unilateral breast cancer	Canalicular carcinoma	ER (+), PR (+) and Her2/neu (+)	24	Yes	10	Breast cancer. Prostatic cancer	1^st^ and 2^nd^	41
Patient 31	41	Female	Unilateral breast cancer	Multifocal, canalicular carcinoma	ER (+), PR (+) and Her2/neu (−)	39	Yes	5	Breast and gastric cancer	1^st^ and 2^nd^	38
Patient 32	46	Female	Ovarian cancer	Endometrioid carcinoma	Not reported	42	Yes	4	Breast cancer. Ovarian cancer	1^st^ and 2^nd^	30
Patient 33	61	Female	Unilateral breast cancer	Canalicular carcinoma	ER (+), PR (+) and Her2/neu (−)	56	Yes	3	Breast cancer (Father with breast cancer)	1^st^ and 2^nd^	31
Patient 34	28	Female	Unilateral breast cancer	Canalicular carcinoma	ER (+), PR (+) and Her2/neu (−)	27	NA (no contact with family)	NA	NA	NA	-
Patient 35	52	Female	Unilateral breast cancer + NF-1	Canalicular carcinoma	ER positive, PR (+)and Her2/neu (−)	46	Yes	2	Breast cancer. Liver cancer	1^st^	49
Patient 36	60	Female	Unilateral breast cancer + NF-1	Canalicular carcinoma	Triple negative	49	Yes	2	Breast cancer. Liver cancer	1^st^	46
Patient 37	40	Female	Unilateral breast cancer	Canalicular carcinoma	Triple negative	36	No	-	-	-	-
Patient 38	39	Female	Unilateral breast cancer	Canalicular carcinoma	Triple negative	35	No	-	-	-	-
Patient 39	44	Female	Bilateral breast cancer	Canalicular carcinoma	Triple negative	1st: 27, 2nd: 33	Yes	1	Breast and colorectal cancer	1^st^	54

ER  =  estrogen receptor; PR  =  progesterone receptor; HER2/neu  =  human epidermal growth factor receptor 2.

NA: Information not available.

NF-1: Neurofibromatosis type 1

**Table 4:** Detection of BRCA deleterious mutations in patients.

Sample	Gene	Mutation	Type of mutation	Position (aa)	Stop position (aa)	Coverage^1^	Clinical relevance	BIC reported	References
Patient 3	*BRCA1*	c.3124_3133delAGCAATATTA	F	1042	1047	77	Yes	No	Not reported
Patient 39	*BRCA1*	c.2805_2808del AGAT	F	935	998	21	Yes	No	Not reported
Patient 1	*BRCA2*	c.5114_5117delTAAA	F	1705	1710	70	Yes	Yes	[53]
Patient 15	*BRCA2*	c.2639_2640delTG	F	880	888	29	Yes	No	Not reported

1 Number of reads per nucleotide.

2 Types of mutations: F: frameshift; S: stop.

**Table 5:** Variants of uncertain significance (VUS) detected in patients.

Patient	Gene	Localization	Variant	Type of Mutation1^1^	Clinical Relevance	PolyPhen2 prediction^2^	BIC Reported
2, 26, 31, 33	*BRCA2*	Exon 27	p.I3412V	M	VUS	B	Yes
5, 26, 30	*BRCA1*	Exon 11	p.Q356R	M	VUS	PD	Yes
6	*BRCA2*	Exon 11	p.H1561N	M	VUS	PD	Yes
6	*BRCA2*	Exon 11	p.V2138F	M	VUS	B	Yes
7	*BRCA1*	Exon 11	p.S1040N	M	VUS	B	Yes
10, 13, 14, 18, 26, 27, 28, 30, 31, 39	*BRCA1*	Intron 1	c.−19T>C	Ts	VUS	-	Yes
10, 11, 17	*BRCA1*	Exon 11	p.K1183R	M	Not reported	B	No
10, 15, 16, 17	*BRCA2*	Intron 11	c.6841+80del TTAA	D	VUS	-	Yes
12, 17	*BRCA1*	Intron 7	c.442−34C>T	Ts	VUS	-	Yes
12	*BRCA1*	Exon 11	p.D1344G	M	VUS	PD	Yes
16	*BRCA2*	Exon 11	p.T1915M	M	VUS	B	Yes
17	*BRCA1*	Exon 12	p.K1489E	M	Not reported	B	No
18, 20, 39	*BRCA1*	Intron 12	c.4097−141A>C	Tv	VUS	-	Yes
18	*BRCA1*	Intron 14	c.4485−64C>G	Tv	VUS	-	Yes
19, 20, 21, 24, 27, 28, 31	*BRCA2*	Exon 15	p.I2490T	M	VUS	B	Yes
21	*BRCA1*	Exon 23	p.V1810V	S	Not reported	B	No
30	*BRCA1*	Exon 23	p.V1804D	M	VUS	B	Yes
30	*BRCA2*	Exon 11	p.S1733S	S	VUS	B	Yes
30	*BRCA2*	Exon 21	K2950N	M	VUS	PD	Yes

1 D: deletion; M: missense mutation; S: synonimous mutation; Ts: transition; Tv: transvertion.

2 B: benign; PD: probably damaging.

## Discussion

Molecular genetic testing of germline mutations in BRCA genes is not common in public institutions in Latin America due to its high costs and limitations in infrastructure. Current protocols for BRCA mutation detection are time consuming and laborious, which makes difficult their implementation in developing countries. Also, the polymorphic nature of BRCA genes, their long size and lack of hot mutation spots highlight the necessity to implement new high throughput diagnostic methodologies. Almost 10% of breast cancer is associated to hereditary mutations [15]. Likewise, the lifetime risk of developing breast cancer is been reported as high as 80% and 50% for *BRCA1* and *BRCA2* mutation carriers, respectively; although it varies between different populations and ethnicities [16], [17]. In this light, BRCA genetic testing is of major diagnostic relevance not only because it provides a clinical preventive approach to family members before the development of cancer, but also can imply novel treatment strategies for affected patients, such as the use of poly-(ADP–ribose) polymerase inhibitors [18]–[20]. Additionally, BRCA genetic tests are central for the determination of founder mutations, which are frequent deleterious variants that can be screened in the population in first-line directed studies to reduce costs and accelerate diagnosis [21], [22]. In the Mexican population no founder mutations have been described.

In these work we analysed BRCA full exome and splice site mutations by massive parallel pyrosequencing. In the evaluation of the method, we found all the mutations present in previously characterized positive controls; negative controls showed no variants. The coverage of the sequences for the mutations varied from 41 to 485X, with quality scores of 20–40 in 95% of the reads throughout all the exonic and splice sites regions. These results led us to evaluate mutations in patients with hereditary breast and ovarian cancer syndrome and in patients with clinical features suggestive for BRCA deleterious mutations. In these analyses we found 4 (10.2%) BRCA mutations in the 39 patients, which is very similar to the prevalence reported by other studies of families with hereditary cancer in Latin America [13], [23], [24]. All the mutations found in these patients have not been previously described and are not reported in the Breast Cancer Information core (BIC) and NCBI variant databases, which is in concordance with the polymorphic nature of these genes [25]. Interestingly, one of these mutations was in a patient with no history of familial cancer, but with strong suggestive clinical manifestations of a BRCA mutation, such as early-onset breast cancer [26]. This result highlights the necessity to extend the screening for BRCA mutations also to candidate patients with no history of familial cancer, which is in concordance with reports that described that 30–50% of BRCA mutation carriers have not family history of breast and ovarian cancer [27], [28]. Remarkably, we found 10 clinically asymptomatic *BRCA2* mutation (c.5114_5117delTAAA) carriers in family 1, which reflects the incomplete penetrance associated with different BRCA mutations and that there are other risk factors associated with the penetrance of BRCA mutations [29]–[32]. In this study we used massive parallel pyrosequencing because its capacity to screen the whole exonic and splice site regions of *BRCA1* and *BRCA2* in up to 8 samples per run and its high depth of sequence, which provides more sensitivity for mutation detection than conventional Sanger sequencing and makes this strategy cost-effective [33]. Also, these advantages offer great benefit to the diagnostic scenario, comparing to other methods. However, this technology has intrinsic limitations, namely the detection of whole exon deletions and the identification of mutations in homopolymeric tracts longer than 6 bases. Since the frequency of exon deletion and large genomic rearrangements is population-dependent and has been described as 1–30% in BRCA-associated cancers, it is determinant to further evaluate putative BRCA mutation-negative samples by complementary methods, such as Multiplex Ligation-dependent Probe Amplification analysis [34]–[36]. Also the evaluation of homopolymeric tract variants, which comprise 12 stretches longer than 6 nt in the *BRCA1* and *BRCA2* coding sequences, should be assessed with alternative methods such as high-resolution-melting-curve-analysis [37]. When negative, these analyzes would rule out the BRCA etiology of the tumor. Thus, in these patients with clear familial history of cancer, the evaluation of mutations in other genes, like *PALB2*, *CHEK2* and *RAD51C*, should also be considered [38]–[41]. This could be the case of some of the families of this study, in which we screened 35 patients with a clear familial history of cancer, but we only found 3 patients with mutations in BRCA. Additionally, the presence of VUS could be related to pathogenic effects at the level of mRNA processing, stability, translation and protein function, as has been described in *BRCA1* and other genes [42]–[46]. The effect of VUS is subject of great interest as their presence exceeds mutations in patients with familial cancer; however, their functional evaluation is far from being a common diagnostic practice. In this regard, the functional evaluation of some VUS in the BRCA genes has showed that single nucleotide variations in introns can influence mRNA processing, producing exon skipping and aberrant out of frame mRNA forms [14]. We found 16 not previously described VUS, especially in patients without deleterious BRCA variants and 4 were predicted to be pathogenic by computational analyses. Functional studies must be undertaken to evaluate their effects. In this concern, we foresee that new routine methods will soon be accessible to determine the molecular and pathological relevance of these variants.

In summary, this work illustrates how hole exonic and splice site massive parallel pyrosequencing can be used as a diagnostic strategy to determine BRCA mutations. Its use circumvents the laborious and time-consuming efforts of the current methodologies. With this technology we found 4 mutations and 16 VUS in our series of patients with familial cancer, which highlights the relevance of this approach as a diagnostic tool and suggests it could be used as a routine practice in public health institutions.
